# The association between the oral microbiome and oral Kaposi's sarcoma-associated herpesvirus (KSHV) shedding is modified by age and HIV status in a rural Ugandan cohort

**DOI:** 10.1080/20002297.2026.2737698

**Published:** 2026-09-29

**Authors:** Katherine R Sabourin, Joseph Mugisha, Vickie A Marshall, Wendell J Miley, Tsute Chen, Jill Billodeaux, Denise Whitby, Rosemary Rochford, Robert Newton

**Affiliations:** a Department of Environmental and Public Health Sciences, University of Cincinnati, Cincinnati, OH, USA; b MRC/UVRI and LSHTM Uganda Research Unit, Entebbe, Uganda; c Viral Oncology Section, AIDS and Cancer Virus Program, Frederick National Laboratory for Cancer Research, Frederick, MD, USA; d The ADA Forsyth Institute, Somerville, MA, USA; e Department of Immunology and Microbiology, University of Colorado, Aurora, CO, USA; f Department of Health Sciences, University of York, York, United Kingdom

**Keywords:** Kaposi sarcoma-associated herpesvirus (KSHV), microbiome, oral, human immunodeficiency virus (HIV), age

## Abstract

**Background:**

Kaposi's sarcoma-associated herpesvirus (KSHV) spreads through saliva. KSHV shedding is intermittent, suggesting individual-level factors affect reactivation.

**Objective:**

Identify associations between oral microbiome and KSHV shedding.

**Design:**

Oral mouthwash samples from 67 KSHV-seropositive participants (ages 4–78) were tested for KSHV DNA by qPCR and microbiome composition via 16S rRNA V4 region sequencing (Illumina MiSeq, Earth Microbiome Project protocols). Samples were selected on age, self-reported HIV status, and KSHV shedding. Alpha and beta diversity and relative abundances at species, genus and phylum levels were compared by KSHV shedding status. Analyses of KSHV shedding and the oral microbiome were stratified by HIV status (*n* = 49 adults only) and by age (*n* = 37 without HIV only, children <18 vs adults ≥18).

**Results:**

Neither alpha nor beta diversity differed by KSHV shedding status. Shedders had higher differential abundance of *Prevotella multisaccharivorax*, *Bifidobacterium dentium, B. moukalabense* and *Bifidobacterium* genus and lower *Absconditabacteria G-1 bacterium HMT-875.* Whether stratified by HIV and KSHV shedding or by age and KSHV shedding, alpha and beta diversity and several taxa at species, genus and phylum levels differed significantly.

**Conclusions:**

Differences in oral microbiome were associated with KSHV shedding and possibly modified by age and HIV status. Future research should consider the multifactorial nature of the oral environment and KSHV reactivation.

## Key messages

- Unstratified analysis revealed no significant differences in alpha or beta diversity between KSHV oral shedders and non-shedders, though differential abundance analysis identified several significantly different taxa.
- Stratification by HIV status and age uncovered significant differences in both alpha and beta diversity, as well as additional differentially abundant taxa, highlighting the importance of host covariates on the relationship between KSHV shedding and the oral microbiome.
- Dysbiosis of the oral microbiome appears to be associated with KSHV reactivation in the oral cavity, with age and HIV status further influencing this relationship, underscoring the need for future research to consider the multifactorial nature of the oral environment in efforts to reduce KSHV transmission and prevent KS.

Kaposi's sarcoma (KS) remains a prevalent cancer in Sub-Saharan Africa (SSA), especially among people living with HIV (PLWH), despite widespread use of antiretroviral therapy (ART). The virus responsible for KS, Kaposi's sarcoma-associated herpesvirus (KSHV), is transmitted primarily via oral fluids. Notably, the detection of KSHV DNA in oral fluids is often intermittent and KSHV viral loads can fluctuate over time, suggesting that individual-level factors influence reactivation, leading to shedding [1–6]. One such factor gaining considerable attention is the role of the human microbiome.

In adult PLWH in the United States, differences in oral microbiome composition were associated with KSHV shedding and oral KS lesions [7,8]. In addition, pathogens such as *P. gingivalis* and *S. aureus*, which are found in the oral cavity, were shown to induce KSHV reactivation *in vitro* [9,10], suggesting that specific species within the oral microbiome may affect oral KSHV reactivation and shedding. Several bacteria-related factors have also been hypothesized to reactivate KSHV either directly or indirectly, including bacterial-derived short-chain fatty acids, lipoteichoic acid and lipopolysaccharides. Reactivation of KSHV has also been hypothesized to occur through bacterial activation of inflammatory cytokines [11]. The results of these previous studies suggest that the oral microbiome may be an important component of KSHV reactivation in the oral cavity.

However, previous studies examining the relationship between the oral microbiome and KSHV shedding have been conducted either *in vitro* or in populations in the United States. While these studies provide important evidence supporting an interaction between the oral microbiome and KSHV shedding, their findings may not be generalizable to other geographic settings, as differences in diet and environmental exposures are known to shape oral microbiome compositions [12,13]. In sub-Saharan Africa, KSHV seroprevalence is highest in the world, with sustained transmission continuing even in the era of widespread ART, yet studies examining the oral microbiome in relation to KSHV in this region are lacking. In Uganda, high rates of KSHV DNA detection in oral fluids have been reported, with both an increased likelihood of detection and higher KSHV viral loads in children compared to adults [6,14–16]. Yet, no single factor has been identified that can explain persistent KSHV reactivation in the oral cavity in these settings, suggesting that it is driven by a multifactorial process. To better understand the oral factors that contribute to KSHV shedding and transmission, this study examined whether the oral microbiome composition is associated with oral KSHV shedding in a cohort of rural Ugandans across the age range.

## Methods

### Study population

70 KSHV seropositive children (age < 18 years) and adults (age 18+ years) were identified from a household cohort study of KSHV shedding carried out in rural Uganda from July to December 2021 [6]. The participants were chosen based on HIV status and KSHV shedding status and were matched by sex and age. At enrolment, a venous blood and mouthwash rinse sample from each individual was collected. Data on age, sex and HIV status were provided through self-report. HIV status was confirmed using previously collected study data for a subset of individuals. All blood and saliva samples were stored at −80 °C until further analysis.

### KSHV serology

At enrolment, up to 5 ml of venous blood was collected in EDTA tubes. Separated plasma was tested by multiplex bead-based assay for the detection of anti-KSHV antibodies to 25 recombinant proteins (K3, K5, K6, K8, K11, K8.1, K10.5, K14, ORF11, ORF18, ORF19, ORF2, ORF20, ORF25, ORF26, ORF33, ORF34, ORF35, ORF37, ORF38, ORF43, ORF44, ORF45, ORF50, ORF52, ORF55, ORF59, ORF6, ORF60, ORF61, ORF63, ORF65, ORF69, ORF72 and ORF73) [17]. The median fluorescence intensity (MFI) across all the counted beads was computed by subtracting the background fluorescence. Plasma collected from US-based adults with active or history of KSHV-associated disease and KSHV DNA detected in peripheral blood mononuclear cells (PBMC) were included as positive controls. Plasma from healthy US-based adult blood donors at low risk of KSHV were included as negative controls. Cut-offs for each antigen were calculated by receiver operating curves using negative control sera by plate [17]. Individuals were identified as KSHV seropositive if IgG to any of 25 KSHV antigens were detected in the plasma.

### KSHV shedding

Mouthwash rinse samples were separated into supernatant and cell pellets. The cell pellets were tested for the detection of KSHV DNA using qPCR [18]. DNA was extracted from samples using the Qiagen blood mini kit (Qiagen, Valencia, CA). DNA was tested using primers specific to the KSHV K6 gene region and the human endogenous retrovirus 3 (ERV-3) gene, which is used for cell quantification determination [19]. For both assays, samples were run in triplicate, and the average values of the independent reactions were calculated. KSHV viral copies were reported as copies per million cell equivalents. KSHV shedding was defined as the detection of KSHV DNA in saliva.

### DNA isolation and next generation sequencing

In mouthwash rinse samples, the V4 region of the 16S rRNA gene was amplified using 515F (GTGCCAGCMGCCGCGGTA) and 806R (GGACTACHVGGGTWTCTAAT) primers and the AccuStart II PCR SuperMix (Quantabio) with equal amounts of DNA from each sample. Construction of primers and amplification procedures followed the Earth Microbiome Project guidelines (www.earthmicrobiome.org) [20]. Amplified DNA was quantified in a PicoGreen assay (Thermo Fisher Scientific), and equal quantities of DNA from each sample were pooled. The pooled DNA was sequenced using a v2 2 × 250 reagent kit on the Illumina MiSeq platform (San Diego, CA) at the Anschutz Center for Microbiome Excellence (ACME).

### Bioinformatics analysis of sequence data

Amplicon sequence variants (ASVs) generated by DADA2 were subjected to species-level taxonomy assignment based on the approach developed by Al-Hebshi et al. [21] against the FOMC Reference Sequence Set (https://microbiome.forsyth.org/ftp/refseq), which consists of 1,015 full-length 16S rRNA sequences from HOMD V15.22, 356 from MOMD V5.1, and 22,126 from NCBI, for a total of 23,497 sequences. The species-level taxonomy assignment algorithm is available at https://github.com/tsute/FOMC_16S_rRNA_Taxonomy_Assignment_Algorithm. Altogether, these sequences represent a total of 17,035 oral and non-oral microbial species. Species-level read count tables were imported using R's ‘phyloseq’ package (version 1.28.0) [22] for downstream analyses. Alpha diversity was calculated from three measurements: 1) number of species (observed), 2) Shannon index and 3) Simpson index using the ‘phyloseq’ package's ‘plot_richness’ function [22]. Alpha diversity significance tests were evaluated with QIIME2 (version 2020.11) ‘alpha-group-significance’ function [23]. Beta diversity NMDS plots were generated with the ‘ordinate’ function in ‘phyloseq’, and beta diversity significance tests were evaluated with QIIME2 ‘beta-group-significance’ function [23]. ANCOMB-BC2 (Analysis of Compositions of Microbiomes with Bias Correction) (version 2.0.2) was used to test the significance of differential abundance by centered log ratio (CLR) between test groups [24]. *p*-values for differential abundance significance were adjusted for multiple tests with the Holm method [25] provided in the ANCOMB-BC2 package and 0.05 was used as the significance cutoff.

Alpha diversity, beta diversity and differential abundances were compared in KSHV shedders and non-shedders. These metrics were also compared by KSHV shedding status by age (adults 18+ years of age vs children < 18 years of age) and HIV serostatus. There were no PLWH under 18 years of age, and analyses of HIV infection included adults 18+ years only. Comparisons of age and KSHV shedding excluded all individuals with HIV. Analyses were adjusted for multiple comparisons. *p*-values < 0.05 were considered statistically significant.

### Ethical approval

Ethical approval was obtained from the Uganda Virus Research Institute Research Ethics Committee (UVRI-REC), Uganda National Council for Science and Technology (UNCST) [GC/127/19/09/745], London School of Hygiene & Tropical Medicine (LSHTM) [17958] and Colorado Multiple Institutional Review Board (COMIRB) [19-0540]. All participants provided written informed consent. Parent/guardian consent was obtained from participants aged < 18 years. Assent was obtained from the children aged 8–17 years. Procedures were completed following the ethical standards of the Helsinki Declaration.

## Results

### Participant characteristics

Of 70 mouthwash rinse samples, 3 were considered unreliable and excluded because of read counts <5,000. Species with <0.1% of total read counts were considered very low-abundant species and excluded. The remaining 67 participants ranged in age from 4 to 78 years and included 49 (73%) adults (aged 18+ years), 37 (55%) females, 30 (61%) PLWH and 30 (45%) KSHV shedders (Table 1).

**Table 1.** Participant characteristics by KSHV shedding status.

	KSHV non-shedder (*n* = 37)	KSHV shedder (*n* = 30)	Total (*n* = 67)
**Age** – mean (range)	37.7 (4–78)	33.6 (4–72)	35.9 (4–78)
Adults (18+ years)	28 (75.7)	21 (70.0)	49 (73.1)
Children (<18 years)	9 (24.3)	9 (30.0)	18 (26.9)
**Sex**			
Female	21 (56.8)	16 (53.0)	37 (55.2)
Male	16 (43.2)	14 (47.0)	30 (44.8)
**HIV status (adults only) *n* = 49**			
With HIV	18 (64.3)	12 (57.1)	30 (61.2)
Without HIV	10 (35.7)	9 (42.9)	19 (38.8)

### Oral microbiome sequencing composition

Mouthwash sample sequences resulted in 3,487,967 reads, with 3,277,508 assigned and used for analysis. 1,404,539 reads were assigned to a single species, and 1,872,969 to multiple species. There were 273 species identified (193 single and 80 multi-species), 97 genera, 45 families, 30 orders, 20 classes and 9 phyla.

Nineteen species had >1% average relative abundance. The most common included *Streptococcus* multi-species (18 species) (19.6%), *Gemella haemolysans, G. morbillorum, G. sanguinis* and *G. sp MOT-043* (5.3%), *Haemophilus parainfluenzae* (5.1%), and *Neisseria cinerea, N. flavescens, N. perflava* and *N. subflava* (5.1%) (Table S2). Fifteen genera had >1% average relative abundance, with the most common being *Streptococcus* (27.9%), *Prevotella* (8.6%), *Haemophilus* (7.3%), *Neisseria* (5.9%) and *Gemella* (5.3%).

### Oral microbiome by KSHV shedding status

Neither alpha nor beta diversity differed at the species (Figure 1), genus, or phylum level (Figure S1) by KSHV shedding status. The differential abundance of *Absconditabacteria G-1 bacterium HMT-875* was lower and *Prevotella multisaccharivorax*, *Bifidobacterium dentium, B. moukalabense* (Figure 2) and *Bifidobacterium* genus were higher in KSHV shedders compared to non-shedders (Figure S2). No differences were identified at the phylum level.

**Figure 1.**
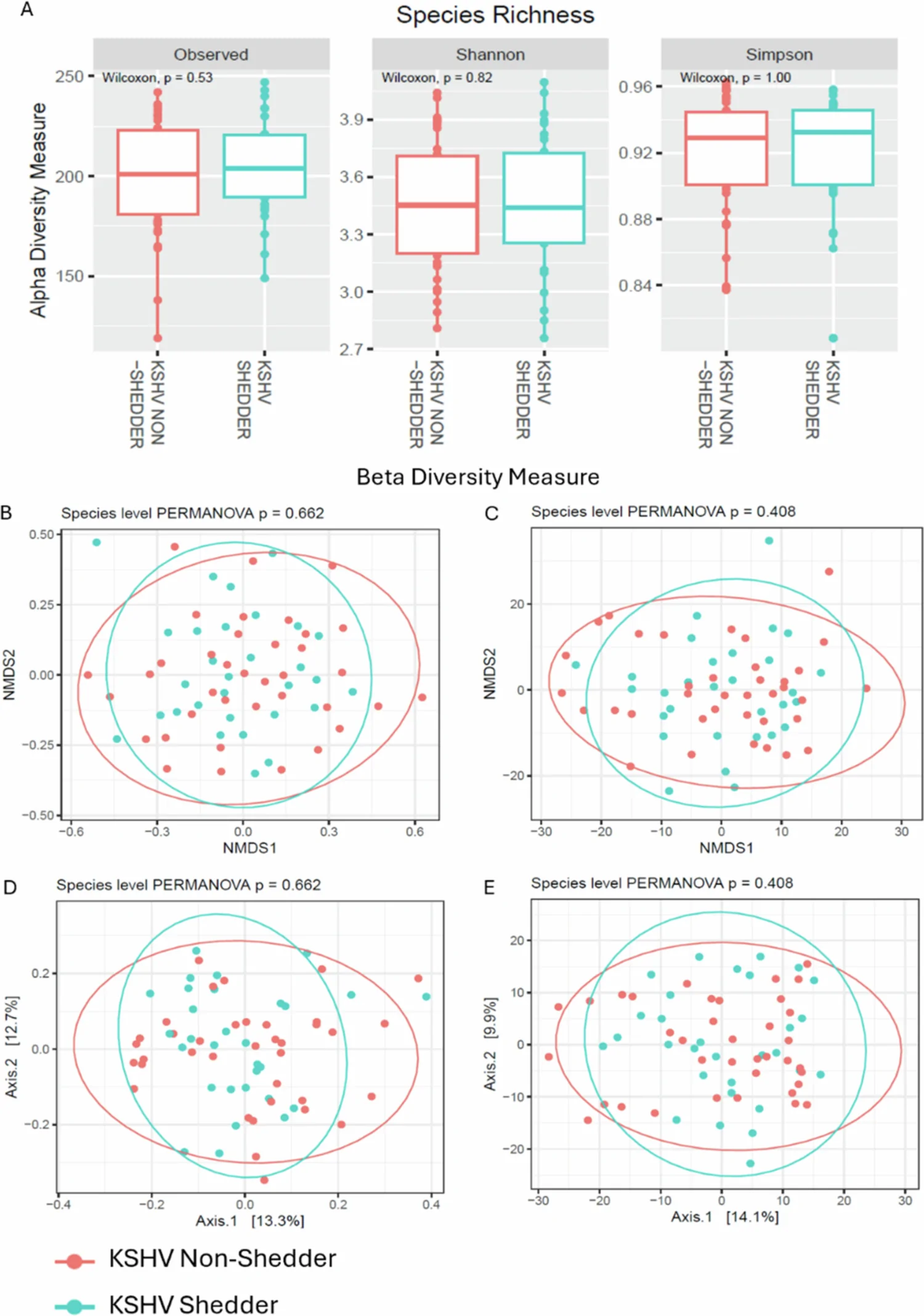
Comparison of oral microbiome at species level by KSHV shedding status among adults (*n* = 67). Measures of oral microbiome were compared by KSHV shedding status (shedders vs non-shedders) and included (a) alpha diversity; (b) NMDS plot with Bray‒Curtis dissimilarity metric; (c) NMDS CLR plot with Aitchison (Euclidean) distance metric; (d) PCoA plot with Bray‒Curtis dissimilarity metric and (e) PCoA CLR plot with Aitchison (Euclidean) distance metric.

**Figure 2.**
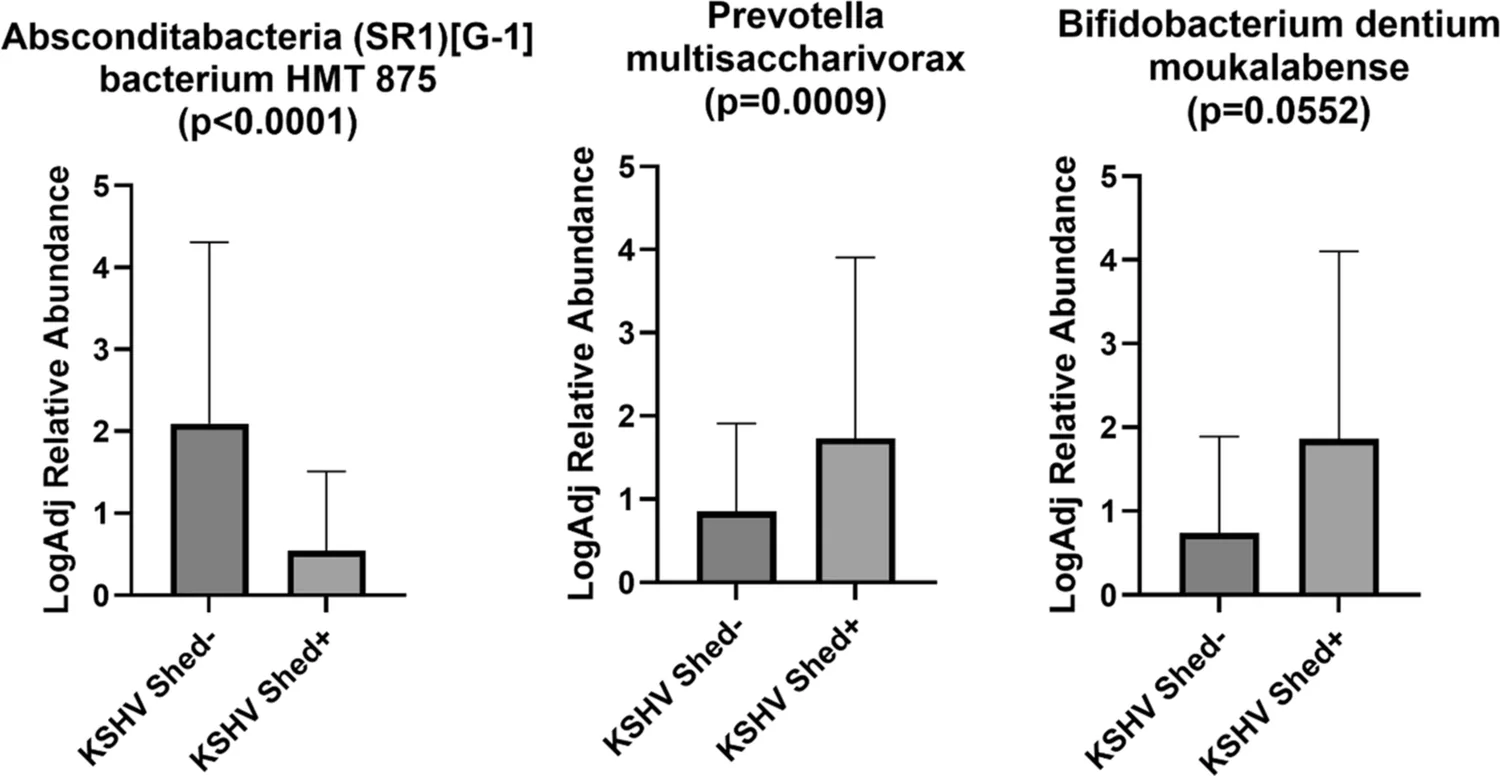
Differential abundance of species by KSHV shedding status (shedders vs non-shedders) (*n* = 67) with significant pairwise comparisons. *p*-values were adjusted with the Holm method [25].

### Oral microbiome by KSHV shedding and HIV status

To determine if the oral microbiome and KSHV shedding relationship is modified by HIV, analyses were restricted to adults and stratified by HIV status. Among adults, species-level alpha diversity was marginally statistically significantly different by HIV infection and KSHV shedding status by Simpson index (*p* = 0.052) (Figure 3A). Genus-level alpha diversity did not differ, but the phylum-level alpha diversity was significantly different between the groups, with the highest in KSHV non-shedders without HIV and the lowest in KSHV non-shedder PLWH (Figure S3). These trends were consistent when measured by the Simpson index, though with differing levels of statistical significance. Beta diversity was statistically significantly different when compared across all four groups at the species (Figure 3B–E), genus and phylum levels (Figure S3).

**Figure 3.**
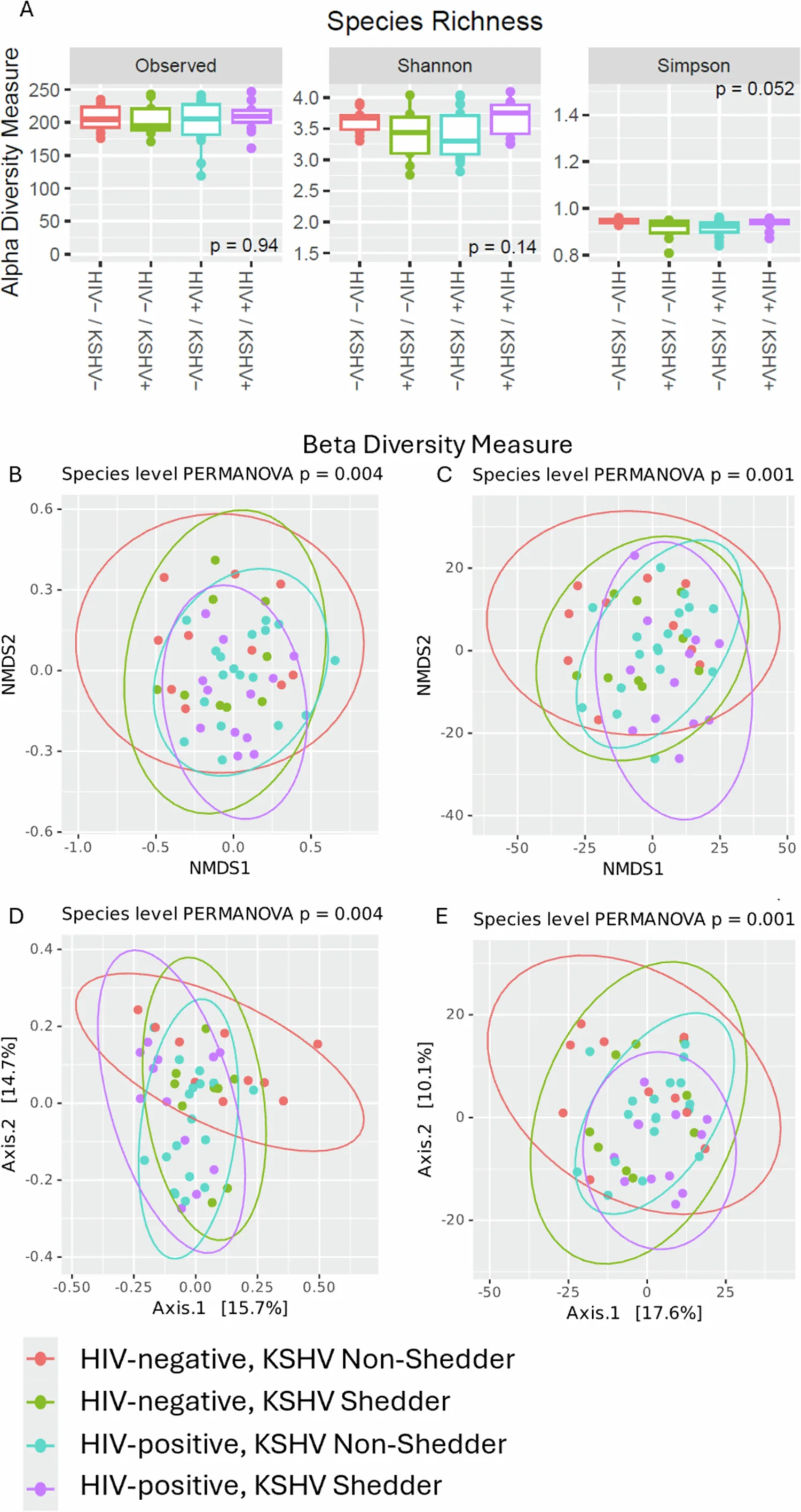
Comparison of oral microbiome at species level by HIV status and KSHV shedding status among adults (*n* = 49). Measures of oral microbiome were compared by HIV status (with vs without HIV) and KSHV shedding status (shedders vs non-shedders) in adults (18+ years of age) and included (a) alpha diversity; (b) NMDS plot with Bray‒Curtis dissimilarity metric; (c) NMDS CLR plot with Aitchison (Euclidean) distance metric; (d) PCoA plot with Bray‒Curtis dissimilarity metric and (e) PCoA CLR plot with Aitchison (Euclidean) distance metric.

Several species-level differences in differential abundance were identified across HIV and KSHV shedding status (Figure 4). KSHV shedders with HIV exhibited lower relative abundance of *Absconditabacteria G-1 HMT 875* and *Limosilactobacillus* but higher *Actinomyces* multi-species (6 species), *Bulleidia extructa*, *Moraxella bovoculi, M.equi, M. lacunata, M. HMT 276*, *Peptidiphaga gingivicola*, *Prevotella multisaccharivorax* and *Romboutsia timonensis* compared to KSHV-non-shedders without HIV. Among PLWH, KSHV shedders had lower relative abundance of *Absconditabacteria G-1 HMT-875* but higher *Prevotella multiforms*, *P. multisaccharivorax* and *Treponema amylovorum* compared to non-shedders. Among individuals without HIV, KSHV shedders exhibited lower relative abundance of *Cloacibacterium HMT-206* compared to non-shedders. Additionally, KSHV shedders without HIV had lower relative abundances of *Cloacibacterium HMT-206* compared to KSHV non-shedders with HIV.

**Figure 4.**
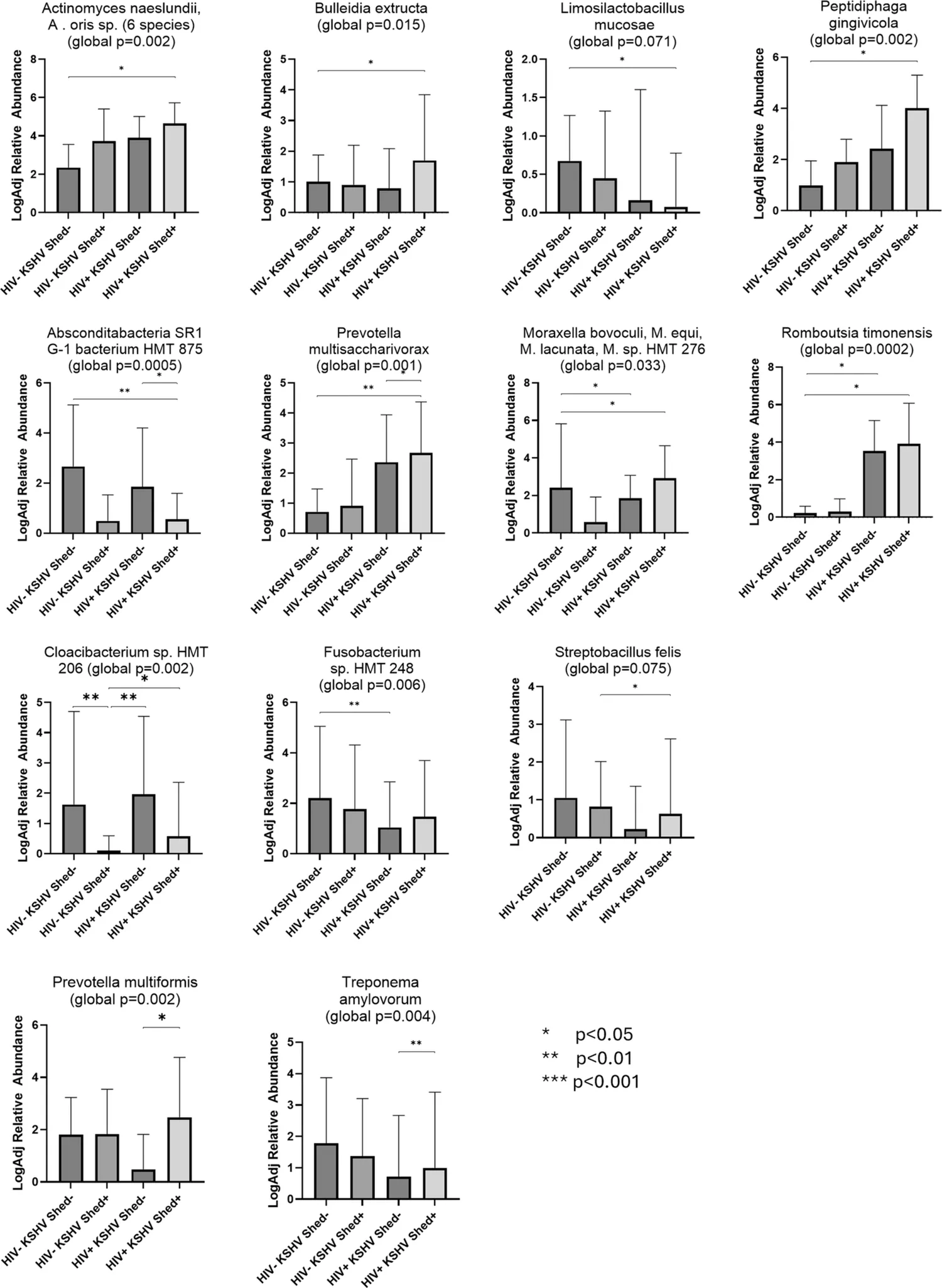
Differential abundance of species by HIV status (with vs without HIV) and KSHV shedding status (shedders vs non-shedders) among adults (*n* = 49) with significant pairwise comparisons. *p*-values were adjusted with the Holm method [25].

Among KSHV shedders, *Cloacibacterium HMT-206* was more abundant, and *Streptobacillus felis* was less abundant in people living with versus without HIV (Figure 4). Among KSHV non-shedders, *Fusobacterium HMT 248* and *Moraxella bococuli, M. equi, M. lacunata* and *M. HMT-276* were less abundant and *R. timonensis* was more abundant in people living with versus without HIV. Several species demonstrated significant global differences without significant pairwise comparisons, including *Actinomyces johnsonii, A. naeslundii, A. HMT-170, A. oris* (clade HMT-171)*, Bacteroidetes G5 HMT-511, Fusobacterium hwasookii, F. HMT-370, F. nucleatum subspecies, Animalis simiae, Lautropia dentalis, L. mirabilis, Peptostreptococcaceae [G-9][Eubacterium] brachy, Prevotella fusca, P. melaninogenica, P. scopos, P. HMT-314, Rothia aeria* and *Streptococcus sanguinis* (Figure S4).

At the genus level, *Cloacibacterium* was less abundant in KSHV shedders without HIV compared to KSHV non-shedders, regardless of HIV status (Figure S5). *Moraxella* was significantly less abundant in KSHV shedders with HIV compared to non-shedders without HIV. Among KSHV non-shedders, *Corynebacterium*, *Fretibacterium*, *Peptidiphaga*, *Peptostreptococcaceae G-9* and *Romboutsia* were more abundant, while *Moraxella* was less abundant in PLWH compared to HIV-negative individuals. *Streptobacillus* was less abundant in KSHV shedders compared to non-shedders among PLWH. Additional genera with significant global trends but no pairwise differences included *Abiotrophia*, *Absconditabacteria G-1*, *Actinomyces*, *Bulleidia*, *Gemella*, *Limosilactobacillus* and *Scardovia*.

Finally, at the phylum level, among KSHV non-shedders, *Synergistetes,* renamed as Synergistota in 2021 [26], was significantly less abundant in participants with versus without HIV (Figure S6). *Absconditabacteria* (*Candidatus Absconditibacteriota*) and *Fusobacteria* (*Fusobacteriota*) showed significant global trends across the four groups but no pairwise differences, suggesting KSHV shedding and HIV infection are associated with oral microbiome composition, potentially in an interdependent manner.

### Oral microbiome by KSHV shedding status and age

To identify if the relationship between the oral microbiome and KSHV shedding is modified by age, analyses were restricted to people living without HIV and stratified by children (<18) versus adults (18+). Significant differences in alpha diversity by Shannon and Simpson indices were observed at the species (Figure 5A), genus, and phylum levels by age and KSHV shedding (Figure S7). Alpha diversity was highest in adult KSHV non-shedders and lowest in child KSHV non-shedders, with both child and adult KSHV shedders exhibiting intermediate diversity, regardless of taxonomy level.

**Figure 5.**
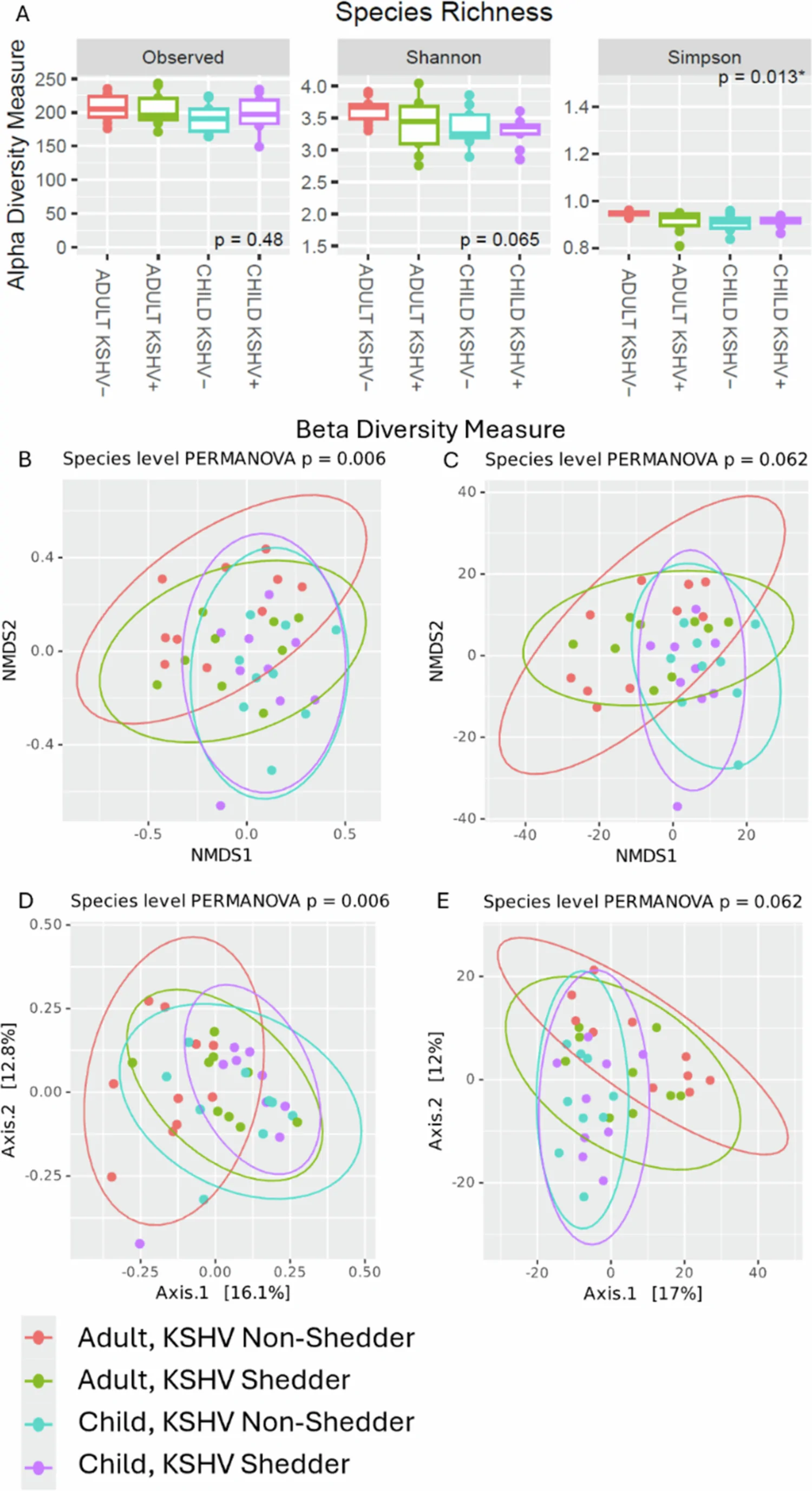
Comparison of oral microbiome at species level by age and KSHV shedding status among those without HIV (*n* = 37). Measures of oral microbiome were compared by age (adults age 18+ years vs children age <18 years) and KSHV shedding status (shedders vs non-shedders) in those without HIV and included (a) alpha diversity; (b) NMDS plot with Bray‒Curtis dissimilarity metric; (c) NMDS CLR plot with Aitchison (Euclidean) distance metric; (d) PCoA plot with Bray‒Curtis dissimilarity metric and (e) PCoA CLR plot with Aitchison (Euclidean) distance metric.

Oral microbiome beta diversity varied by age and KSHV shedding status at the species level (Figure 5B-E) but did not reach statistical significance when assessed by Aitchison (Euclidean) distance metric (*p* = 0.062). At the genus level, beta diversity differed significantly across groups regardless of the distance metric, whereas phylum-level differences were modest and reached only borderline significance when measured using the Bray–Curtis dissimilarity metric (*p* = 0.07) (Table S6).

Distinct differences in relative abundance were observed by KSHV shedding and age at the species level. Among children, KSHV shedders exhibited higher relative abundances of *Bifidobacterium dentium, B. moukalabense* and lower *Cryptobacterium curtum* and *Prevotella aurantiaca* compared to non-shedders (Figure 6). In adults, KSHV shedders showed reduced relative abundance of *Cloacibacterium HMT-206* relative to non-shedders. Furthermore, child KSHV shedders had lower relative abundances of *Absconditabacteria G-1 HMT-345*, *Cryptobacterium curtum*, *Prevotella aurantiaca* and *Treponema HMT-256* compared to adult KSHV non-shedders. Among KSHV shedders, *Cloacibacterium HMT-206* and *Cryptobacterium curtum* were more abundant in children than in adults. Conversely, among non-shedders, the relative abundance of *Fusobacterium HMT-248* and *Porphyromonas gingivalis* were lower in children than in adults. Statistically significant trends were detected across the four groups for *Actinomyces naeslundii, A. oris* (six species), *Corynebacterium matruchotii*, *Kingella HMT-012*, *Lautropia mirabilis*, *Leptotrichia shahii*, *Peptidiphaga gingivicola*, *Peptostreptococcus stomatis* and *Treponema medium HMT-237*; however, no pairwise comparisons reached statistical significance (Figure S8).

**Figure 6.**
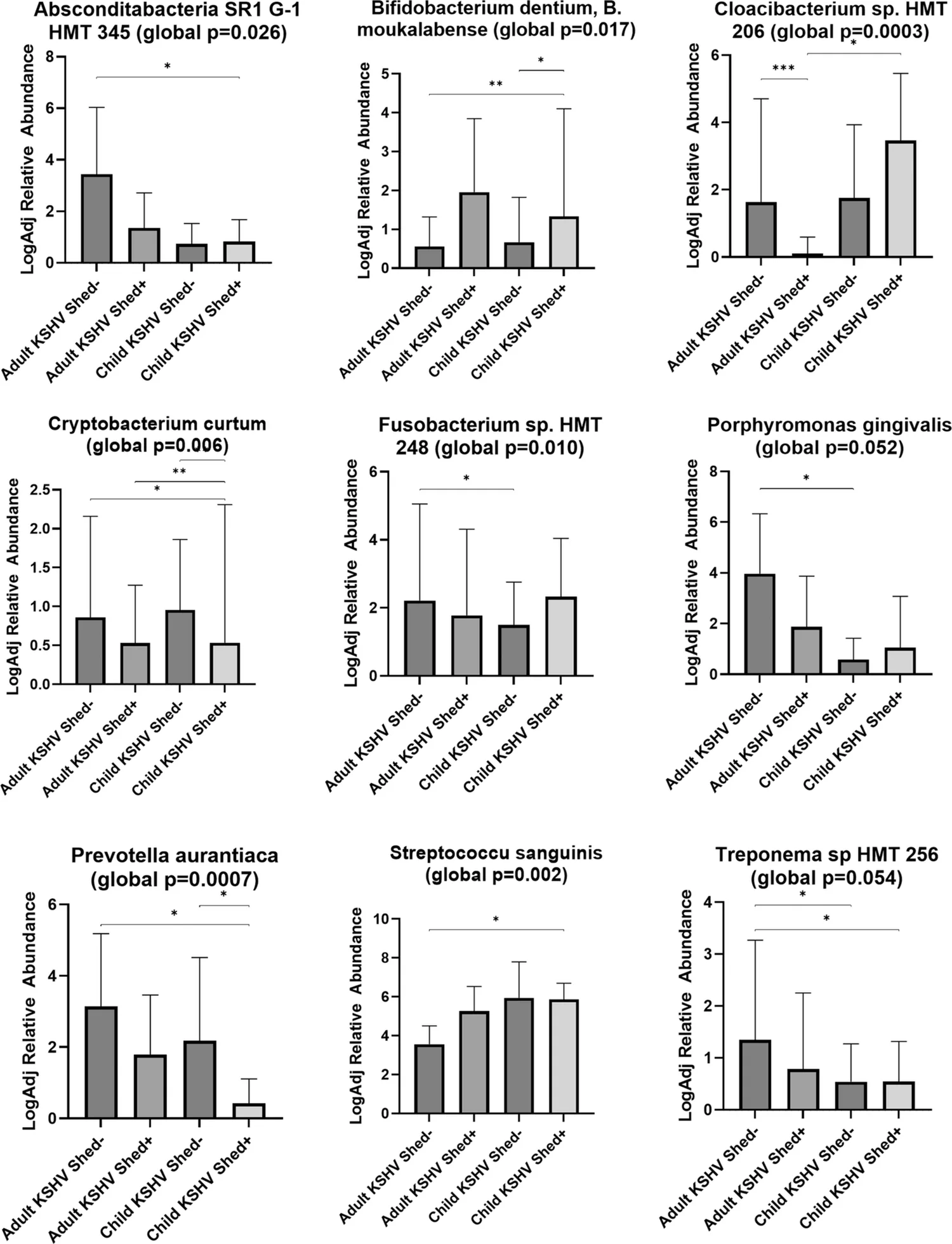
Differential abundance of species by age (adults age 18+ years vs children age < 18 years) and KSHV shedding status (shedders vs non-shedders) in those without HIV (*n* = 37) with significant pairwise or global comparisons. *p*-values were adjusted with the Holm method [25].

At the genus level, notable differences in relative abundance were also observed by age and KSHV shedding status. Among children, KSHV shedders exhibited higher *Bifidobacterium* and lower *Cryptobacterium* compared to non-shedders (Figure S9). In adults, KSHV shedders had reduced relative abundance of *Cloacibacterium* versus non-shedders. Child KSHV shedders had higher *Bifidobacterium* and lower *Cryptobacterium* relative abundance than adult KSHV non-shedders. Additionally, child KSHV non-shedders showed higher *Mitsuokella* and lower *Peptostreptococcus* relative abundance compared to adult KSHV shedders. Within KSHV shedders, children had higher relative abundance of *Cloacibacterium* and *Cryptobacterium* and lower *Peptostreptococcus* than adults. Significant trends were observed for *Corynebacterium*, *Kingella*, *Lautropia* and *Peptidiphaga*, but no pairwise comparisons reached statistical significance. At the phylum level, no statistically significant differences were detected by age and KSHV shedding status. These findings highlight differences in oral microbiome composition associated with both age and KSHV shedding status.

## Discussion

Our primary goal was to identify oral microbial features associated with oral KSHV shedding in a rural Ugandan population with a high burden of KSHV seroprevalence. Multiple species and genera within the oral microbiome were associated with oral KSHV shedding in this rural Ugandan population. In addition, alpha and beta diversities and several species, genera and phyla were different by KSHV shedding when also stratified by HIV status or age. These findings provide novel data on the composition of the oral microbiome in relation to oral KSHV shedding from a rural Sub-Saharan African population and are further strengthened by the inclusion of both individuals without HIV and children, groups that have been underrepresented in prior studies. These findings support a potential relationship between the oral microbiome and oral KSHV reactivation, which may be modified by HIV status and age, which is consistent with the multifactorial nature of factors affecting oral KSHV reactivation.

No differences in alpha or beta diversity were observed by KSHV shedding status. However, higher relative abundances of *P. multisaccharivorax* and *B dentium/B. moukalabense,* and the genus *Bifidobacterium* were identified in KSHV shedders. Interestingly, *P. multisaccharivorax* has previously been identified in endodontic abscesses, and both *P. multisaccharivorax*, *B dentium* and *Bifidobacterium* have been associated or correlated with dental caries [27–31]. Reduced relative abundances of *Absconditabacteria G-1 bacterium HMT-875* were also found in KSHV shedders. Although the role of *Absconditabacteria (SR1)* in the oral microbiome remains poorly understood, it has been detected in multiple oral sites, including the healthy oral cavity, suggesting a possible association with oral microbial homeostasis [32,33]. Together, these findings suggest a potential association between the oral microbiome and KSHV shedding that may, in part, reflect differences in oral health status or inflammatory states. It is also possible that oral health status may have contributed to the observed associations. Unfortunately, information on oral health conditions was not collected in this study, preventing the assessment of whether factors such as dental caries or other oral diseases influence the findings.

When stratified by HIV status among adults, differences in alpha and beta diversity and species-level differential abundances were observed by KSHV shedding. Although the overall trends in alpha diversity varied by KSHV shedding and HIV status, the pairwise comparisons did not reach statistical significance. These findings differ from those of a study of adult PLWH in the United States, which reported lower oral microbiota alpha diversity in KSHV-seropositive shedders compared to KSHV seronegative non-shedders [7]. In contrast, the analyses presented here included only KSHV-seropositive individuals. A lower alpha diversity has also been observed in PLWH with oral KS compared to those with KSHV DNA in saliva but no oral KS [8]. A study in Xinjiang, China, reported no differences in salivary microbiota alpha diversity but significant differences in beta diversity between KSHV-seropositive and -seronegative PLWH, though oral shedding was not assessed [34]. In this data, PLWH who shed KSHV tended to have higher alpha diversity, whereas among individuals without HIV, the opposite trend was observed, with higher alpha diversity in non-shedders. These findings are consistent with previous research showing HIV-related differences in oral microbial communities [35]. Understanding this relationship may help clarify the mechanisms of oral KSHV reactivation in PLWH.

It is possible that differences observed in diversity metrics by HIV and KSHV shedding status were driven by specific bacterial taxa. Previous studies have reported genus-level differences in the salivary microbiota, including *Streptococcus*, *Alloprevotella* and *Fusobacterium,* between KSHV seropositive and seronegative PLWH [34]. Another study found that KSHV-seropositive shedders had increased *Firmicutes* and *Streptococcus* and decreased *Lactobacillales* and *Pasteurellaceae* Compared to KSHV seronegative non-shedders [7]. Additionally, several taxa at the species, genus, order and family levels differed in a cohort of HIV/KSHV co-infected individuals with and without oral KS and oral KSHV shedding [8]. Notably, none of these previous studies included participants without HIV and so were unable to assess the effects of HIV infection on the oral microbiome and KSHV shedding.

This study included participants with and without HIV and was able to examine differential abundances of several species and genera that varied based on KSHV shedding and HIV infection status. Among PLWH, *T. amylovorum*, *P. multiformis* and *P. multisaccharivorax*, which have been linked to inflammatory oral health disorders, including periodontitis, candidiasis and/or dental caries [27–29,36–38], were in higher abundance in KSHV shedders where as *Absconditabacteria HMT875* was at lower abundance among shedders, and among people without HIV, shedders had lower *Cloacibacterium HMT206*. Not much is known about this specific species of *Cloacibacterium*, though a previous study found it was positively associated with higher average periodontal pocket probing depth [39], while *Cloacibacterium* genus has been found in healthy larynx [40]. Shifts in the relative abundance of these taxa could indicate an inflammatory oral environment, potentially resulting from oral health disorders or other mechanisms, which may in turn promote KSHV reactivation [41,42]. Furthermore, high production of inflammatory cytokines has been shown to trigger oral KSHV *in vitro* [43,44]. Among PLWH, poorer immune control has been associated with oral manifestations and a potentially inflammatory oral environment [45–48]. In this study, CD4 counts, HIV viral loads and durations of antiretroviral therapy were not collected, limiting the ability to assess HIV viral suppression or immune status, though all participants living with HIV reported current ART use.

Differences in microbiome composition by KSHV shedding status were also identified when stratified by age group. Age is an important determinant of KSHV shedding, with several studies in Uganda reporting higher shedding frequencies and viral loads among children compared to adults [6,14–16]. In this analysis, significant differences in alpha and beta diversity and several differentially abundant species and genera were identified by KSHV shedding status and age. Notably, adult non-shedders exhibited the highest alpha diversity, while child non-shedders had the lowest, with KSHV shedders in both age groups falling in between, suggesting that the oral microbiome of KSHV shedders may be more similar across ages than to that of non-shedders. These findings highlight a novel aspect of KSHV epidemiology. Given that the oral microbiome changes with age, with children typically having less diverse microbiome profiles than adults [49–51], it is possible that specific microbiome profiles or particular taxa in children promote oral KSHV reactivation. *B. dentium*/*moukalabense,* linked with caries as described above [28,30], were identified in higher abundance in child KSHV shedders compared to non-shedders, while *Cryptobacterium curtum* and *P. aurantiaca* were in lower abundances. And in adults *C. HMT-206* was in lower abundance in shedders, similar to what was seen among adults living with HIV in this cohort. This novel data is the first of its kind to examine oral microbiome in the context of KSHV shedding in both children and adults and can be used to inform future investigations into the mechanisms involved. Other factors that shape the oral microbiome may influence these findings, such as diet, oral hygiene, oral health, alcohol and tobacco consumption, and co-infections and should be included in future research on KSHV shedding.

Overall, these findings point towards differences in oral microbial community composition between KSHV shedding groups. Although no clear pattern in the differential abundance of taxa emerged by KSHV shedding status, several of the taxa associated with shedding have previously been linked to oral health or oral inflammatory disorders, supporting the plausibility of a relationship between the oral microbial environment and KSHV activity. Future studies should collect oral health data via clinical examination, as this is a key confounder for oral microbiome studies. In addition, the observed modification of associations by age and HIV status suggests that host factors may influence microbiome-virus interactions, highlighting the complexity of the oral ecosystem.

The results of this study support a potential association between the oral microbiome and oral KSHV shedding in a rural African population, though the results should be interpreted with caution. The algorithm used an alignment method that enables accurate species assignment. However, by using 16S rRNA gene sequencing, this study may not be able to discriminate closely related bacterial species within a genus or species complex [52,53]. Therefore, the observed associations may reflect broader shifts at the genus or community level. Future studies that incorporate broader coverage approaches, such as shotgun metagenomic sequencing, meta-transcriptomics, or cultivation-based methods, will be important for clarifying the functional role(s) of the oral microbiome in KSHV reactivation. In addition, the use of 16S sequencing does not provide information on microbial functional activity, limiting the ability to evaluate the biological mechanisms underlying the observed associations. The cross-sectional design of the study also prevents determination of whether shifts in the oral microbiome contribute to KSHV shedding or occur as a downstream effect of viral replication and associated immune responses. The study also could not assess oral microbiome changes over time and how these changes may correlate with KSHV shedding, which is known to be intermittent [1–6]. Longitudinal studies will be needed to disentangle the complex interactions between the oral microbiome, host immune status and KSHV reactivation.

## Conclusions

In a rural African population, where the global burden of KSHV seroprevalence and KS incidence is highest, this study offers important insights into how the oral microbiome may be related to oral KSHV shedding. Dysbiosis of the oral microbiome appears to be associated with KSHV shedding in the oral cavity, with age and HIV status contributing to the observed differences in this relationship. Future research on oral KSHV reactivation should consider the multifactorial nature of the oral environment, which can inform strategies aimed at reducing KSHV transmission and prevent KS development worldwide.

## Supplementary Material

HAKU_OralMicro and KSHV_Supplement.docx

## Data Availability

The datasets generated and/or analyzed during the current study are not publicly available in accordance with the rules and regulations of MRC/UVRI but are available from the corresponding author upon reasonable request.
