# Supplementary material for: The association between the oral microbiome and oral Kaposi's sarcoma-associated herpesvirus (KSHV) shedding is modified by age and HIV status in a rural Ugandan cohort
Source: J Oral Microbiol. 2026 Sep 29;18(1):2737698. doi: 10.1080/20002297.2026.2737698 (PMC13629773; doi:10.1080/20002297.2026.2737698)
Supplement: Supplementary file 1 [file ZJOM_A_2737698_SM4922.docx]

Table S1. Most common species detected by average relative abundance

| Species | Average % relative abundance |
| --- | --- |
| *Streptococcus cristatus downii gordonii gwangjuense infantis inf* ...(18 species) | 19.6% |
| *Gemella haemolysans morbillorum sanguinis sp. MOT-043* | 5.3% |
| *Haemophilus parainfluenzae* | 5.1% |
| *Neisseria cinerea flavescens perflava subflava* | 5.1% |
| *Morococcus Neisseria cerebrosus flava macacae mucosa sicca* | 3.6% |
| *Rothia mucilaginosa* | 3.5% |
| *Granulicatella elegans* | 2.3% |
| *Veillonella atypica dispar* | 2.2% |
| *Streptococcus salivarius thermophilus vestibularis* | 2.0% |
| *Aggregatibacter segnis sp. HMT 458 sp. HMT 512* | 1.7% |
| *Granulicatella adiacens* | 1.7% |
| *Streptococcus australis parasanguinis parasanguinis clade 411 pa* ...(7 species) | 1.7% |
| *Haemophilus haemolyticus influenzae seminalis sp. HMT 036 sp.* ...(5 species) | 1.6% |
| *Fusobacterium periodonticum* | 1.6% |
| *Prevotella melaninogenica* | 1.5% |
| *Streptococcus gordonii himalayensis sp. HMT 056* | 1.3% |
| *Streptococcus cristatus cristatus clade 578 cristatus clade 886 ...*(4 species) | 1.3% |
| *Lautropia mirabilis* | 1.2% |
| *Alloprevotella sp. HMT 473* | 1.1% |
| Other species | 36.6% |

Table S2. Most common genera detected by average relative abundance

| Genus | Average % relative abundance |
| --- | --- |
| *Streptococcus* | 27.9% |
| *Prevotella* | 8.6% |
| *Haemophilus* | 7.3% |
| *Neisseria* | 5.9% |
| *Gemella* | 5.3% |
| *Veillonella* | 4.6% |
| *Fusobacterium* | 4.4% |
| *Rothia* | 4.4% |
| *Granulicatella* | 4.0% |
| *Morococcus_Neisseria* | 3.6% |
| *Leptotrichia* | 3.1% |
| *Porphyromonas* | 2.4% |
| *Aggregatibacter* | 2.2% |
| *Alloprevotella* | 1.9% |
| *Lautropia* | 1.5% |
| Other genus | 12.9% |

Beta Diversity Measure

D

E

B

C

A

KSHV Non-Shedder

KSHV Shedder

D

E

B

C

Beta Diversity Measure

A

D

E

KSHV Non-Shedder

KSHV Shedder

**Figure S1. Comparison of oral microbiome at genus and phylum level by KSHV Shedding Status (n=67).** Measures of oral microbiome were compared by HIV status (negative vs positive) and KSHV Shedding Status (shedders vs non-shedders) in adults (18+ years of age) and included A) Alpha diversity; B) NMDS Plot with Bray-Curtis Dissimilarity Metric; C) NMDS CLR Plot with Aitchison (Euclidean) Distance Metric; D) PCoA Plot with Bray-Curtis Dissimilarity Metric; E) PCoA CLR Plot with Aitchison (Euclidean) Distance Metric

**Figure S2.** Differential abundance of genus by KSHV shedding status (shedders vs non-shedders) among adults (n=67) with significant pairwise comparisons.

**Figure S3.** Comparison of oral microbiome at genus and phylum level by HIV status and KSHV shedding status among adults (n=49). Measures of oral microbiome were compared by HIV status (with vs without HIV) and KSHV shedding status (shedders vs non-shedders) in adults (18+ years of age) and included A) Alpha diversity; B) NMDS Plot with Bray-Curtis Dissimilarity Metric; C) NMDS CLR Plot with Aitchison (Euclidean) Distance Metric; D) PCoA Plot with Bray-Curtis Dissimilarity Metric; E) PCoA CLR Plot with Aitchison (Euclidean) Distance Metric

Table S3. Universal and Pairwise Statistics for alpha diversity measures of oral microbiome by HIV infection status and KSHV shedding status among adults only.

*Alpha Diversity comparisons by Kruskal-Wallis between*

| **Observed Features** |  | H | p-value | q-value |
| --- | --- | --- | --- | --- |
| Overall Comparison |  | 0.382065 | 0.944 |  |
| Group 1 | Group 2 |  |  |  |
| HIV-Neg NON-SHEDDER (n=10) | HIV-Neg SHEDDER (n=9) | 0.081954 | 0.775 | 0.869 |
|  | HIV-Pos NON-SHEDDER (n=18) | 0.147288 | 0.701 | 0.869 |
|  | HIV-Pos SHEDDER (n=12) | 0.027205 | 0.869 | 0.869 |
| HIV-Neg SHEDDER (n=9) | HIV-Pos NON-SHEDDER (n=18) | 0.264955 | 0.607 | 0.869 |
|  | HIV-Pos SHEDDER (n=12) | 0.031627 | 0.859 | 0.869 |
| HIV-Pos NON-SHEDDER (n=18) | HIV-Pos SHEDDER (n=12) | 0.217087 | 0.641 | 0.869 |

| **Shannon Index** |  | H | p-value | q-value |
| --- | --- | --- | --- | --- |
| Overall Comparison |  | 5.468517 | 0.141 |  |
| Group 1 | Group 2 |  |  |  |
| HIV-Neg NON-SHEDDER (n=10) | HIV-Neg SHEDDER (n=9) | 1.500000 | 0.221 | 0.331 |
|  | HIV-Pos NON-SHEDDER (n=18) | 2.503448 | 0.114 | 0.227 |
|  | HIV-Pos SHEDDER (n=12) | 0.213043 | 0.644 | 0.773 |
| HIV-Neg SHEDDER (n=9) | HIV-Pos NON-SHEDDER (n=18) | 0.023810 | 0.877 | 0.877 |
|  | HIV-Pos SHEDDER (n=12) | 2.909091 | 0.088 | 0.227 |
| HIV-Pos NON-SHEDDER (n=18) | ADULT HIV-Pos SHEDDER (n=12) | 3.313620 | 0.069 | 0.227 |

| **Simpson Index** |  | **H** | **p-value** | **q-value** |
| --- | --- | --- | --- | --- |
| Overall Comparison |  | 7.738585 | 0.052 |  |
| Group 1 | Group 2 |  |  |  |
| HIV-Neg NON-SHEDDER (n=10) | HIV-Neg SHEDDER (n=9) | 3.526667 | 0.060 | 0.153 |
|  | HIV-Pos NON-SHEDDER (n=18) | 5.519540 | 0.019 | 0.113 |
|  | HIV-Pos SHEDDER (n=12) | 0.213043 | 0.644 | 0.773 |
| HIV-Neg SHEDDER (n=9) | HIV-Pos NON-SHEDDER (n=18) | 0.000000 | 1.000 | 1.000 |
|  | HIV-Pos SHEDDER (n=12) | 2.671717 | 0.102 | 0.153 |
| HIV-Pos NON-SHEDDER (n=18) | HIV-Pos SHEDDER (n=12) | 3.012545 | 0.083 | 0.153 |

Table S4. Universal and Pairwise Statistics for beta diversity measures of oral microbiome by HIV infection status and KSHV shedding status among adults only.

*Beta Diversity by Pairwise permanova results*

| **Bray-Curtis** |  | Sample size | Permutations | pseudo-F | p-value | q-value |
| --- | --- | --- | --- | --- | --- | --- |
| Overall Comparison |  | 49 | 999 | 1.883 | **0.002*** |  |
| Group 1 | Group 2 |  |  |  |  |  |
| HIV-Neg NON-SHEDDER | HIV-Neg SHEDDER | 19 | 999 | 1.527249 | 0.113 | 0.170 |
|  | HIV-Pos NON-SHEDDER | 28 | 999 | 3.069575 | **0.001*** | **0.006*** |
|  | HIV-Pos SHEDDER | 22 | 999 | 2.309065 | **0.015*** | **0.045*** |
| HIV-Neg SHEDDER | HIV-Pos NON-SHEDDER | 27 | 999 | 1.245847 | 0.205 | 0.205 |
|  | HIV-Pos SHEDDER | 21 | 999 | 1.695590 | **0.027*** | 0.054 |
| HIV-Pos NON-SHEDDER | HIV-Pos SHEDDER | 30 | 999 | 1.308822 | 0.173 | 0.205 |

| **Correlation** |  | Sample size | Permutations | pseudo-F | p-value | q-value |
| --- | --- | --- | --- | --- | --- | --- |
| Overall Comparison |  | 49 | 999 | 2.219968 | **0.012*** |  |
| Group 1 | Group 2 |  |  |  |  |  |
| HIV-Neg NON-SHEDDER | HIV-Neg SHEDDER | 19 | 999 | 1.778030 | 0.168 | 0.336 |
|  | HIV-Pos NON-SHEDDER | 28 | 999 | 3.816488 | **0.009*** | **0.042*** |
|  | HIV-Pos SHEDDER | 22 | 999 | 3.311926 | **0.014*** | **0.042*** |
| HIV-Neg SHEDDER | HIV-Pos NON-SHEDDER | 27 | 999 | 0.291608 | 0.837 | 0.837 |
|  | HIV-Pos SHEDDER | 21 | 999 | 1.390438 | 0.251 | 0.377 |
| HIV-Pos NON-SHEDDER | HIV-Pos SHEDDER | 30 | 999 | 0.999053 | 0.380 | 0.456 |

| **Aitchison** |  | Sample size | Permutations | pseudo-F | p-value | q-value |
| --- | --- | --- | --- | --- | --- | --- |
| Overall Comparison |  | 49 | 999 | 1.690271 | **0.003*** |  |
| Group 1 | Group 2 |  |  |  |  |  |
| HIV-Neg NON-SHEDDER | HIV-Neg SHEDDER | 19 | 999 | 1.136523 | 0.265 | 0.318 |
|  | HIV-Pos NON-SHEDDER | 28 | 999 | 2.118958 | **0.010*** | **0.024*** |
|  | HIV-Pos SHEDDER | 22 | 999 | 2.335188 | **0.009*** | **0.024*** |
| HIV-Neg SHEDDER | HIV-Pos NON-SHEDDER | 27 | 999 | 1.056452 | 0.335 | 0.335 |
|  | HIV-Pos SHEDDER | 21 | 999 | 1.483649 | 0.066 | 0.099 |
| HIV-Pos NON-SHEDDER | HIV-Pos SHEDDER | 30 | 999 | 1.815116 | **0.012*** | **0.024*** |

**Figure S4.** Differential abundance of species by HIV status (with vs without HIV) and KSHV shedding status (shedders vs non-shedders) among adults (n=49) with significant global comparisons.

**Figure S5.** Differential abundance of genus by HIV status (with vs without HIV) and KSHV shedding status (shedders vs non-shedders) among adults (n=49) with significant pairwise or global comparisons.

**Figure S6.** Differential abundance of phylum by HIV status (with vs without HIV) and KSHV shedding status (shedders vs non-shedders) among adults (n=49) with significant pairwise or global comparisons.

**Figure S7.** Comparison of oral microbiome at genus and phylum levels by age and KSHV shedding status among those without HIV (n=37). Measures of oral microbiome were compared by age (adults age 18+ years vs children age <18 years) and KSHV shedding status (shedders vs non-shedders) in those without HIV and included A) Alpha diversity; B) NMDS Plot with Bray-Curtis Dissimilarity Metric; C) NMDS CLR Plot with Aitchison (Euclidean) Distance Metric; D) PCoA Plot with Bray-Curtis Dissimilarity Metric; E) PCoA CLR Plot with Aitchison (Euclidean) Distance Metric

Table S5. Universal and Pairwise Statistics for alpha diversity measures of oral microbiome by Age (children <18 years vs adults 18+ years) and KSHV shedding status among participants without HIV infection only.

*Alpha Diversity comparisons by Kruskal-Wallis between*

| **Observed Features** |  | H | p-value | q-value |
| --- | --- | --- | --- | --- |
| Overall Comparison |  | 2.485501 | 0.478 |  |
| Group 1 | Group 2 |  |  |  |
| ADULT NON-SHEDDER (n=10) | ADULT SHEDDER (n=9) | 0.081954 | 0.775 | 0.775 |
|  | CHILD NON-SHEDDER (n=9) | 2.537226 | 0.111 | 0.667 |
|  | CHILD SHEDDER (n=9) | 0.375329 | 0.540 | 0.715 |
| ADULT SHEDDER (n=9) | CHILD NON-SHEDDER (n=9) | 1.032254 | 0.310 | 0.715 |
|  | CHILD SHEDDER (n=9) | 0.281282 | 0.596 | 0.715 |
| CHILD NON-SHEDDER (n=9) | CHILD SHEDDER (n=9) | 0.563353 | 0.453 | 0.715 |

| **Shannon Index** |  | H | p-value | q-value |
| --- | --- | --- | --- | --- |
| Overall Comparison |  | 7.229493 | 0.065 |  |
| Group 1 | Group 2 |  |  |  |
| ADULT NON-SHEDDER (n=10) | ADULT SHEDDER (n=9) | 1.500000 | 0.221 | 0.441 |
|  | CHILD NON-SHEDDER (n=9) | 4.506667 | 0.034 | 0.101 |
|  | CHILD SHEDDER (n=9) | 7.706667 | 0.006 | **0.033*** |
| ADULT SHEDDER (n=9) | CHILD NON-SHEDDER (n=9) | 0.001949 | 0.965 | 0.965 |
|  | CHILD SHEDDER (n=9) | 0.563353 | 0.453 | 0.679 |
| CHILD NON-SHEDDER (n=9) | CHILD SHEDDER (n=9) | 0.017544 | 0.895 | 0.965 |

| **Simpson Index** |  | H | p-value | q-value |
| --- | --- | --- | --- | --- |
| Overall Comparison |  | 10.747368 | 0.013* |  |
| Group 1 | Group 2 |  |  |  |
| ADULT NON-SHEDDER (n=10) | ADULT SHEDDER (n=9) | 3.526667 | 0.060 | 0.121 |
|  | CHILD NON-SHEDDER (n=9) | 6.406667 | **0.011*** | **0.034*** |
|  | CHILD SHEDDER (n=9) | 10.140000 | **0.001*** | **0.009*** |
| ADULT SHEDDER (n=9) | CHILD NON-SHEDDER (n=9) | 0.235867 | 0.627 | 0.627 |
|  | CHILD HIV-Neg SHEDDER (n=9) | 0.438596 | 0.508 | 0.627 |
| CHILD HIV-Neg NON-SHEDDER (n=9) | CHILD HIV-Neg SHEDDER (n=9) | 0.329435 | 0.566 | 0.627 |

Table S6. Universal and Pairwise Statistics for beta diversity measures of oral microbiome by Age (children <18 years vs adults 18+ years) and KSHV shedding status among participants without HIV infection only.

*Beta Diversity by Pairwise permanova results*

| **Bray-Curtis** |  | Sample size | Permutations | pseudo-F | p-value | q-value |
| --- | --- | --- | --- | --- | --- | --- |
| Overall Comparison |  | 37 | 999 | 1.605999 | **0.006*** |  |
| Group 1 | Group 2 |  |  |  |  |  |
| ADULT NON-SHEDDER | ADULT SHEDDER | 19 | 999 | 1.527249 | 0.120 | 0.213 |
|  | CHILD NON-SHEDDER | 19 | 999 | 2.390296 | **0.002*** | **0.012*** |
|  | CHILD SHEDDER | 19 | 999 | 2.512274 | **0.004*** | **0.012*** |
| ADULT SHEDDER | CHILD NON-SHEDDER | 18 | 999 | 1.331397 | 0.142 | 0.213 |
|  | CHILD SHEDDER | 18 | 999 | 0.939931 | 0.542 | 0.650 |
| CHILD NON-SHEDDER | CHILD SHEDDER | 18 | 999 | 0.768098 | 0.760 | 0.760 |

| **Correlation** |  | Sample size | Permutations | pseudo-F | p-value | q-value |
| --- | --- | --- | --- | --- | --- | --- |
| Overall Comparison |  | 37 | 999 | 1.829796 | **0.040*** |  |
| Group 1 | Group 2 |  |  |  |  |  |
| ADULT NON-SHEDDER | ADULT SHEDDER | 19 | 999 | 1.778581 | 0.169 | 0.282 |
|  | CHILD NON-SHEDDER | 19 | 999 | 2.387531 | 0.063 | 0.189 |
|  | CHILD SHEDDER | 19 | 999 | 3.153831 | **0.001*** | **0.006*** |
| ADULT SHEDDER | CHILD NON-SHEDDER | 18 | 999 | 1.432791 | 0.272 | 0.326 |
|  | CHILD SHEDDER | 18 | 999 | 1.313297 | 0.188 | 0.282 |
| CHILD NON-SHEDDER | CHILD SHEDDER | 18 | 999 | 0.470047 | 0.848 | 0.848 |

| **Aitchison** |  | Sample size | Permutations | pseudo-F | p-value | q-value |
| --- | --- | --- | --- | --- | --- | --- |
| Overall Comparison |  | 37 | 999 | 1.687928 | **0.004*** |  |
| Group 1 | Group 2 |  |  |  |  |  |
| ADULT NON-SHEDDER | ADULT SHEDDER | 19 | 999 | 1.136277 | 0.264 | 0.317 |
|  | CHILD NON-SHEDDER | 19 | 999 | 2.693871 | **0.003*** | **0.018*** |
|  | CHILD SHEDDER | 19 | 999 | 2.267533 | **0.007*** | **0.021*** |
| ADULT SHEDDER | CHILD NON-SHEDDER | 18 | 999 | 1.766998 | **0.011*** | **0.022*** |
|  | CHILD SHEDDER | 18 | 999 | 1.290170 | 0.130 | 0.195 |
| CHILD NON-SHEDDER | CHILD SHEDDER | 18 | 999 | 0.802212 | 0.811 | 0.811 |

**Figure S8.** Differential abundance of species by age (adults age 18+ years vs children age <18 years) and KSHV shedding status (shedders vs non-shedders) in those without HIV (n=37) with significant global comparisons

**Figure S9.** Differential abundance of genus by age (adults age 18+ years vs children age <18 years) and KSHV shedding status (shedders vs non-shedders) in those without HIV (n=37) with significant pairwise or global comparisons

Table S7. Table summarizing statistically significant differential abundance of species by group comparison.

|  |  | **By KSHV Shedding and HIV Status** | | | | | **By KSHV Shedding and Age** | | | | |
| --- | --- | --- | --- | --- | --- | --- | --- | --- | --- | --- | --- |
| **Species with identified differential abundance** | **KSHV shed vs non** | **Among HIV+, KSHV shed vs non-shed** | **Among HIV-, KSHV shed vs non-shed** | **HIV+/KSHV shed vs HIV-/KSHV non-shed** | **Among KSHV shed, HIV+ vs HIV-** | **Among KSHV non-shed, HIV+ vs HIV-** | **Among <18, KSHV shed vs non-shed** | **Among 18+, KSHV shed vs non-shed** | **<18/KSHV shed vs 18+/KSHV non-shed** | **Among KSHV shed, <18 vs 18+** | **Among KSHV non-shed, <18 vs 18+** |
| *Absconditabacteria HMT-875* | ↓ | ↓ | – | ↓ | – | – | – | – | – | – | – |
| *Absconditabacteria HMT-345* | – | – | – | – | – | – | – | – | ↓ | – | – |
| *Actinomyces spp.* | – | – | – | ↑ | – | – | – | – | – | – | – |
| *Bifidobacterium dentium* | ↑ | – | – | – | – | – | ↑ | – | ↑ | – | – |
| *Bifidobacterium moukalabense* | ↑ | – | – | – | – | – | ↑ | – | ↑ | – | – |
| *Bulleidia extructa* | – | – | – | ↑ | – | – | – | – | – | – | – |
| *Cloacibacterium HMT-206* | – | – | ↓ | – | ↑ | – | – | ↓ | – | ↑ | – |
| *Cryptobacterium curtum* | – | – | – | – | – | – | ↓ | – | ↓ | ↑ | – |
| *Fusobacterium HMT-248* | – | – | – | – | – | **↓** | – | – | – | – | ↓ |
| *Limosilactobacillus mucosae* | – | – | – | ↓ | – | – | – | – | – | – | – |
| *Moraxella spp.* | – | – | – | ↑ | – | **↓** | – | – | – | – | – |
| *Peptidiphaga gingivicola* | – | – | – | ↑ | – | – | – | – | – | – | – |
| *Porphyromonas gingivalis* | – | – | – | – | – | – | – | – | – | – | ↓ |
| *Prevotella aurantiaca* | – | – | – | – | – | – | ↓ | – | ↓ | – | – |
| *Prevotella multiformis* | – | ↑ | – | – | – | – | – | – | – | – | – |
| *Prevotella multisaccharivorax* | ↑ | ↑ | – | ↑ | – | – | – | – | – | – | – |
| *Romboutsia timonensis* | – | – | – | ↑ | – | ↑ | – | – | – | – | – |
| *Streptobacillus felis* | – | – | – | – | ↓ | – | – | – | – | – | – |
| *Streptococcus sanguinis* | – | – | – | – | – | – | – | – | ↑ | – | – |
| *Treponema amylovorum* | – | ↑ | – | – | – | – | – | – | – | – | – |
| *Treponema HMT-256* | – | – | – | – | – | – | – | – | ↓ | – | ↓ |
